# Is Negative Emotion Differentiation Associated With Emotion Regulation Choice? Investigations at the Person and Day Level

**DOI:** 10.3389/fpsyg.2021.684377

**Published:** 2021-07-09

**Authors:** Mia S. O'Toole, Emma Elkjær, Mai B. Mikkelsen

**Affiliations:** Department of Psychology and Behavioural Sciences, Aarhus University, Aarhus, Denmark

**Keywords:** emotion differentiation, emotion regulation, emotion regulation choice, experience- sampling method, emotion granularity

## Abstract

Negative emotion differentiation (ED) has been suggested to be important for adaptive emotion regulation (ER). However, knowledge concerning how ED may impact specific ER strategy choice remains surprisingly sparse. We therefore investigated (1) if person-level negative ED was associated with habitual use of individual ER strategies, (2) how person-level negative ED was associated with daily use of individual ER strategies, and finally (3) how within-person daily fluctuations in negative ED were associated with daily use of individual ER strategies. During a 10-day experience sampling study, 90 healthy participants rated their momentary emotions and their ER efforts in response to those emotions. ER strategies included four putatively adaptive strategies (reflection, distancing, non-reactivity, reappraisal) and four putatively maladaptive strategies (rumination, experiential avoidance, expressive suppression, worry). Results revealed that negative ED at the person level was neither associated with habitual nor daily ER strategy endorsement when controlling for negative emotions. Likewise, associations between within-individual daily variation in negative ED and daily ER did not remain statistically significant after controlling for negative emotions. The results thus point to no or weak associations between negative ED and ER choice above and beyond negative emotions. Future experimental studies addressing ED at the momentary level and teasing out the ED–ER causal timeline are needed to further evaluate ED–ER associations. Findings from such research may represent an important step toward refining psychotherapeutic interventions aimed at improving emotional problems.

## Introduction

Across theoretical positions, emotions are said to arise in the face of something personally relevant in a given situation (Gross and Barrett, 2011). The majority of emotion theories also converge on the idea that one of the most prominent features of emotions is their functionality, evidenced as the enactment of this personal relevance, preparing the individual to approach, or avoid an object (Frijda, 2007; Scherer and Moors, 2019). As such, emotions provide the individual with information about themselves in relation to the world, and point to whether or not their needs and goals are being met (Frijda, 2007; Gross and Barrett, 2011; Gross, 2014). Therefore, the ability to become aware of one's emotions is deemed important for healthy emotional functioning (Lindquist and Barrett, 2010; Grühn et al., 2013; Kashdan et al., 2015; Grossmann et al., 2016b). Individuals may gain such awareness, and thus experience their emotions, in different ways. One indicator of emotion experience that has received considerable theoretical and empirical attention is emotion differentiation (ED). Emotion differentiation refers to the individual's ability to experience distinct emotions (e.g., anxiety, sadness) in a granular manner, independent of each other (Kashdan et al., 2015; Grossmann et al., 2016b). Emotion differentiation has often been operationalized as the inverse consistency between either negative or positive emotions over a number of occasions, typically derived empirically from experience-sampling studies conducted over a set number of days (Kashdan et al., 2015; O'Toole et al., 2020). It has been argued that because ED entails a detailed or rich experience of emotions, the good differentiator will have access to more information about themselves in relation to the world and thus a better foundation upon which to base their subsequent emotion regulatory efforts (Gohm and Clore, 2002; Demiralp et al., 2012; Kircanski et al., 2012; Kuppens and Verduyn, 2015; Mennin and Fresco, 2015; Kalokerinos et al., 2019; Seah and Coifman, 2021; Thompson et al., 2021).

Empirical evidence supports the assumption that ED—in particular negative ED—is associated with overall better mental health (Smidt and Suvak, 2015) and with specific positive outcomes pertaining to situational responding (for a review see Kalokerinos et al., 2019), including reduced alcohol consumption (Kashdan et al., 2010), less impulsivity (Tomko et al., 2015), more empathetic attunement to one's partner (Erbas et al., 2016), and less aggression (Pond et al., 2012). Thus, there appears to be a direct, attenuating effect of ED on a range of maladaptive behaviors. In addition to such direct effects, ED also appears to act as a moderator by attenuating the negative consequences of specific emotion regulation (ER) strategies. For instance, Zaki et al. (2013) used an experience-sampling protocol on individuals with borderline personality disorder and found that the association between rumination and both acts and urges of non-suicidal self-injury was negative for those with higher levels of ED, whereas it was positive for those with lower levels. Starr et al. (2017) investigated ED as a moderator of associations between daily perseverative self-focused ER (i.e., brooding and savoring) and daily depressive symptoms in both young healthy adults and veterans from primary care. Across the two populations, both negative and positive ED moderated the associations between perseverative self-focused strategies and depressive symptoms such that low ED was associated with an enhanced association. Seah et al. (2020) examined if negative ED moderated the positive association between rumination and frequency of social avoidance within the context of social anxiety disorder. Across two studies, negative ED was indeed found to moderate the relationship between rumination and social avoidance. Specifically, they found that the positive association between rumination and social avoidance was significant for low but not moderate to high negative ED. Finally, Liu et al. (2019) conducted a 6-month prospective longitudinal study, examining the moderating role of ED on the association between rumination and depression. They found that ED of positive and negative emotions together interacted with rumination to predict significant changes in depression, after controlling for mean levels of emotion. Together, these studies may point to a protective factor of, in particular, negative ED, such that the negative consequences of a specific ER strategy are weakened when the individual is adept at differentiating their emotional experience.

Knowing how negative ED may alter the effect of specific ER strategies (e.g., rumination) on certain desired or undesired outcomes (e.g., depressive symptoms) is clearly important for honing instances in which improved negative ED may play a role in alleviating clinical conditions or reducing maladaptive behavior. However, little is known about how negative ED influences overall ER choice. The first study to evaluate the association between ED and a variety of ER strategies was conducted in 2001 by Barrett and colleagues. In a 14-day diary study of 53 participants, emotions and ER were rated within the context of the most intense emotional experiences. The study authors hypothesized that the ED–ER relationship would be strongest in this context. This hypothesis was rooted in the assumption that the press for ER is greatest in situations characterized by negative emotions. Specifically, negative emotions are believed to have greater informational value in signaling the need to change or adjust one's current state or activity, and a failure to respond to a negative signal can prevent the individual from taking steps to avoid potential harm (Barrett et al., 2001, p. 715). Consistent with their hypothesis, the authors found that individuals with higher negative ED reported stronger regulatory efforts in response to negative emotions, operationalized as the combined use of nine ER strategies. Kalokerinos et al. (2019) recently extended this research taking into account important limitations. First, Barrett et al. (2001) averaged all ER strategies. However, the individual's habitual use of certain strategies appears to be differentially associated with well-being and healthy functioning. For instance, Aldao et al. (2010) found that habitual use of so-called putatively maladaptive strategies (i.e., rumination, suppression), compared to adaptive strategies (i.e., reappraisal, problem solving), were more strongly associated with psychopathology (i.e., symptoms of anxiety, depression, and eating disorders). Second, the ER strategies in the study by Barrett et al. (2001) were averaged retrospectively according to how much participants indicated that they had used the strategies over the previous 2 weeks. Such assessment method may not capture the dynamics of the individual's choice of ER strategies from moment to moment. Kalokerinos et al. (2019) concluded that a strategy-specific approach, evaluating ER at the momentary level, was needed. Accordingly, they examined how negative ED was related to the momentary selection of a variety of ER strategies in two experience-sampling studies. Contrary to their hypothesis, they found only few relationships between negative ED and the selection of putatively adaptive or maladaptive strategies. However, consistent with the studies mentioned above on the moderating role of ED, they found that among low differentiators, regulatory strategies were more strongly associated with increased negative emotion than they were among high differentiators.

In the studies by Kalokerinos et al. (2019), ED was operationalized as is typically the case, namely as the inverse average consistency [i.e., intra-class correlation (ICC)] between emotion ratings across all measurement occasions for each individual. As such, ED was conceptualized as a matter of individual differences, that is, a stable, person-level variable. However, it is increasingly acknowledged that ED has both stable and variable parts (Tomko et al., 2015; Grossmann et al., 2016b; Erbas et al., 2018; O'Toole et al., 2020). ED may both vary within person from day to day or moment to moment (Erbas et al., 2018), and may even improve overall following psychotherapy (Van Der Gucht et al., 2019; Mikkelsen et al., 2021). Accordingly, ED should be investigated both at the person level, where ED is averaged across all measurement occasions (i.e., between persons), and as fluctuations from this average (i.e., within-person change; Tomko et al., 2015; Erbas et al., 2018). As with ED, ER may also be conceptualized at the person-level, reflecting habitual regulatory tendencies, from which point the individual may fluctuate from day to day or moment to moment (Gross, 2014; Aldao et al., 2015; Kalokerinos et al., 2019). At this point, it remains unknown how ED—at the between and within-person level—may be differentially associated with the use of particular ER strategies. We therefore wanted to add to this rather sparse literature on the ED–ER association, investigating associations between both person-level negative ED and within-person daily deviations from this person-level on the one side, and daily ER on the other.

### The Present Study

This study was an experience-sampling study (involving two samples; *N* = 51 and 39), in which participants were asked to rate both emotions and ER efforts three (i.e., Sample 1) or four (i.e., Sample 2) times a day over 10 days. With this design, we were able to evaluate both the person- and day-level association between negative ED and daily ER strategy choices. At baseline, we also inquired about habitual ER as well as overall positive and negative affect. We employed a strategy-specific approach, exploring (1) if person-level negative ED was associated with habitual use of individual ER strategies. We then explored (2) how person-level negative ED was associated with daily use of individual ER strategies, and finally (3) how within-person daily fluctuations in negative ED were associated with daily use of individual ER strategies. Our first aim serves as an extension of the study by Barrett et al. (2001), exploring the association between negative ED and average ER, however, from a strategy-specific approach. The second and third aim represent an endeavor to follow up on the studies by Kalokerinos et al. (2019) by not only addressing the association between person-level ED and daily choice of ER strategies, but also adding to this research by investigating the association between within-person daily fluctuations in ED and daily ER. For all three aims, we hypothesized that higher negative ED would be positively associated with putatively adaptive ER strategies and negatively associated with putatively maladaptive ER strategies. Following recent findings and recommendations (see Dejonckheere et al., 2019; Kalokerinos et al., 2019), we wanted to assess the unique contribution of ED to ER strategy selection and therefore evaluated the extent to which ED was associated with ER above and beyond negative emotions.

## Methods

### Participants and Procedures

Participants were students recruited from the local university through advertisements on social media and lectures at the university. To participate, individuals had to be above the age of 18 years, proficient in the Danish language, and able to provide written consent to participate. Participants were excluded if they were not able to engage in the daily monitoring procedures over the following 10 days. Written consent was obtained upon participants having received written information about the study where it was underscored that participation was voluntary with no consequences in case of declining to participate or dropping out. After completing a baseline questionnaire, an experience-sampling study was conducted. Participants received three (Sample 1) or four (Sample 2) text messages every day for 10 days containing a link to an online questionnaire. They received the text messages on their personal smart phone. The link and questionnaire was created and distributed via the software SurveyXact. The text messages were sent at random times between 10 a.m. and 9 p.m. in 1-h intervals. The baseline questionnaire had a completion time of 20 to 30 min, whereas the daily measures had one of 2–3 min. Participants were compensated with a gift voucher (250 DKK/app. 40 USD)^1^.

### Measures

#### Baseline Person-Level Measures of Emotions

Baseline negative emotions were measured with seven negative emotion words (i.e., guilty, ashamed, nervous, sad, disgusted, angry, frustrated) and positive emotions were measured with seven positive emotion words (i.e., happy, appreciative, satisfied, amused, curious, proud, enthusiastic). These emotion categories have often used in experience sampling studies (e.g., Kashdan and Steger, 2006; Demiralp et al., 2012; O'Toole et al., 2014; Kashdan et al., 2015). Each emotion was rated on a 5-point Likert Scale (1 = not at all; 5 = very much).

#### Baseline, Person-Level Measures of Emotion Regulation

Eight ER strategies were evaluated. The different strategies were chosen based on (1) obtaining an equal number of putatively adaptive and maladaptive strategies and (2) typically investigated ER strategies (Aldao et al., 2010). The four putatively adaptive strategies included *reappraisal* which was evaluated with the 6-item subscale (rated from 1 to 7) from the Emotion Regulation Questionnaire (ERQ; Gross and John, 2003, α = 0.79), *distancing* which was evaluated with the 11-item subscale (rated from 1 to 5) from the Experiences Questionnaire (EQ; Fresco et al., 2007, α = 0.86), *non-reactivity* which was evaluated with the 7-item non-reactivity subscale (rated from 1 to 5) of the Five Facet Mindfulness Questionnaire (FFMQ; Baer et al., 2008, α = 0.90), and *reflection* which was evaluated with the 12-item reflection subscale (rated from 1 to 5) from the Reflection and Rumination Questionnaire (RRQ; Trapnell and Campbell, 1999, α = 0.93). The putatively maladaptive strategies included *expressive suppression*, measured with the 4-item subscale (rated from 1 to 7) from the ERQ (Gross and John, 2003, α = 0.78), *experiential avoidance* evaluated with the 7-item experiential avoidance subscale (rated from 1 to 7) of the Acceptance and Action Questionnaire (AAQ; Bond and Bunce, 2003, α = 0.56), *worry* which was evaluated with the 16-item Penn State Worry Questionnaire (rated from 1 to 5) (PSWQ; Meyer et al., 1990, α = 0.89/0.83), and *rumination* evaluated with the 12-item rumination subscale (rated from 1 to 5) from the RRQ (Trapnell and Campbell, 1999, α = 0.93).

#### Day-Level Measures

##### Daily Emotions

Daily emotions were assessed with the same seven negative and seven positive emotion words used at baseline (e.g., Kashdan and Steger, 2006; Demiralp et al., 2012; O'Toole et al., 2014; Kashdan et al., 2015). For each emotion, participants rated the degree to which it reflected the way they felt at that moment of the day on a 5-point Likert Scale.

##### Daily ER

Daily ER was measured with items reflecting the eight strategies measured at baseline. Specifically, each strategy was evaluated with two items, which were chosen based on the highest factor loading as obtained in validation studies while considering the ability for the item to be meaningfully repeated within a daily context (cf. Kashdan and Steger, 2006; O'Toole et al., 2017). All items were rated on a 5-point Likert Scale and changed into present tense to assess the extent to which the strategy was employed in the *present moment*. Specifically, participants were instructed to “Think about the emotional experience you just rated. Now rate the extent to which you applied each of the following strategies to handle this emotional experience.” The items were all rated from 1 to 5 and included:

##### Daily Putatively Adaptive Emotion Regulation Strategies

Reflection: “I am exploring my ‘inner' self” and “I am looking at my life in a philosophical perspective” (RRQ; Trapnell and Campbell, 1999). Reappraisal: “I am changing the way I am thinking of the situation” and “I am changing the way I am thinking of my feelings” (ERQ; Gross and John, 2003). Distancing: “I am treating myself kindly” and “I am observing my feelings without being drawn into them” (EQ; Fresco et al., 2007). Non-reactivity: “I am perceiving my feelings and emotions without having to react to them” and “I am noticing thoughts or images without reacting” (FFMQ; Baer et al., 2008).

##### Daily Putatively Maladaptive Emotion Regulation Strategies

Worry: “My worries are overwhelming me” and “I am worrying and can't stop worrying” (PSWQ; Meyer et al., 1990). Rumination: “I am ruminating over or dwelling on things that are happening to me” and “I'm playing back over in my mind how I acted in a past situation” (RRQ; Trapnell and Campbell, 1999). Expressive suppression: “I am controlling my emotions by not expressing them” and “I am keeping my emotions to myself” (ERQ; Gross and John, 2003). Experiential avoidance: “I am afraid of my feelings” and “I am trying to suppress thoughts and feelings that I don't like by just not thinking about them” (AAQ; Bond and Bunce, 2003).

#### Reliability and Validity of Daily ER Strategy Items

Reliability between the two items was evaluated by calculating the correlation coefficient for each individual across the study period. Correlation coefficients (*r*) ≥ 0.5 were taken to be indicative of satisfactory internal reliability. This criterion was met for all ER strategies except distancing and experiential avoidance, which were correlated to a moderate degree (*r*s = 0.3). The validity of the daily ER strategies was evaluated in a multilevel model (MLM), where the baseline measure of the ER strategy served as predictor of the daily measure (see description of MLMs below). Associations of a medium strength (*r* ≥ 0.3) were taken to be indicative of satisfactory validity (Kashdan and Steger, 2006; O'Toole et al., 2014). This criterion was met for all ER strategies except reappraisal (see Table 1). Two sets of analyses were then conducted, serving as further validation of the chosen ER strategies as putatively adaptive (when associated with more positive emotions) and putatively maladaptive (when associated with more negative emotions), including correlation analyses between baseline ER strategies and baseline negative or positive emotions, in addition to MLMs exploring daily associations between person mean-centered daily ER and positive and negative emotions (see description of MLMs below). Results from correlation analyses at baseline, see Table 2, revealed positive and statistically significant correlations between putatively adaptive strategies and positive emotions, and negative and statistically significant correlations for negative emotions, all of a small to medium magnitude. This was with the exception of reflection. Concerning the putatively maladaptive strategies at baseline, worry and rumination showed the expected pattern where correlations were statistically significant and of a small to medium magnitude. However, experiential avoidance was only correlated at the statistically significant level with positive emotions, and although in the expected direction, both correlations were non-significant for expressive suppression. Concerning the daily measures, person mean-centered daily putatively adaptive ER strategies showed statistically significant positive associations with positive emotions of medium and large magnitudes except for reappraisal, where the association was non-significant. Associations with negative emotions were negative, statistically significant and of a large magnitude for distancing and non-reactivity. For reappraisal the association with negative emotions was significant, positive, and of a small to medium magnitude. For reflection, this association was non-significant. All person mean-centered daily putatively maladaptive ER strategies showed statistically significant negative associations with positive emotions of medium and large magnitudes. Associations with negative emotions were all positive, statistically significant, and of medium and large magnitudes. See Table 3.

**Table 1:** Correlation coefficients between the two ER items and associations between baseline and daily measures of ER.

	**Within-person correlation between the two items across the study period**	**Associations between baseline ER and daily ER**
RRQ reflection	0.59	*t* = 6.1, *p* < 0.001, *r* = 0.55
EQ distancing	0.32^*^	*t* = 7.2, *p* < 0.001, *r* = 0.61
FFMQ non-reactivity	0.56	*t* = 6.4, *p* < 0.001, *r* = 0.57
ERQ reappraisal	0.67	*t* = 2.6, *p* = 0.011, *r* = 0.27^*^
RRQ rumination	0.65	*t* = 4.3, *p* < 0.001, *r* = 0.42
AAQ experiential avoidance	0.32^*^	*t* = 4.4, *p* < 0.001, *r* = 0.43
ERQ suppression	0.57	*t* = 7.2, *p* < 0.001, *r* = 0.61
PSWQ worry	0.72	*t* = 6.7, *p* < 0.001, *r* = 0.59

* *Value below threshold of 0.5 (within-person correlation) or 0.3 (association between baseline and daily measure of emotion regulation)*.

*AAQ, Acceptance and Action Questionnaire (Bond and Bunce, 2003); EQ, Experiences Questionnaire (Fresco et al., 2007); ER, emotion regulation; ERQ, Emotion Regulation Questionnaire (Gross and John, 2003), FFMQ, Five Facet Mindfulness Questionnaire (Baer et al., 2008); PSWQ, Penn State Worry Questionnaire (Meyer et al., 1990); RRQ, Rethinking Rumination Questionnaire (Nolen-Hoeksema et al., 2008)*.

**Table 2:** Associations expressed as correlation coefficients between emotion regulation at baseline, person-level emotion differentiation, and negative and positive emotions at baseline (without/with mean levels of negative emotions across the study period as a covariate).

	**Person-level negative ED**	**Positive emotions**	**Negative emotions**
Person-level negative ED	–	0.12	−0.29^**^
Reflection	−0.02/−0.06	0.16	−0.02
Distancing	0.24^*^/0.14	0.43^***^	−0.40^***^
Non-reactivity	0.28^**^/0.09	0.37^***^	−0.34^**^
Reappraisal	0.18/0.08	0.25^*^	−0.29^**^
Rumination	−0.28^**^/−0.07	−0.38^***^	0.47^***^
Experiential avoidance	−0.17/−0.04	−0.22^*^	0.09
Expressive suppression	−0.04/ < 0.01	−0.13	0.19
Worry	−0.30^**^/−0.13	−0.30^**^	0.35^**^
Positive emotions	0.12	–	−0.41^***^
Negative emotions	−0.29^**^	−0.41^***^	–

* *p < 0.05*,

** *p < 0.01*,

*** *p <0.001. ED, emotion differentiation*.

**Table 3:** Associations between person mean-centered daily emotion regulation and daily positive and negative emotions.

**Person mean-centered daily emotion regulation**	**Positive emotions**	**Negative emotions**
Reflection	*t* = 4.6, ***p*** **<** **0.001**, *r* = 0.44	*t* <0.1, *p* = 0.969, *r* = 0.10
Distancing	*t* = 9.5, ***p*** **<** **0.001**, *r* = 0.72	*t* = −6.6, ***p*** **<** **0.001**, *r* = 0.58
Non-reactivity	*t* = 2.7, ***p*** **=** **0.009**, *r* = 0.28	*t* = −4.4, ***p*** **<** **0.001**, *r* = 0.43
Reappraisal	*t* = 0.8, *p* = 0.445, *r* = 0.08	*t* = 2.2, ***p*** **=** **0.035**, *r* = 0.23
Rumination	*t* = −4.3, ***p*** **<** **0.001**, *r* = 0.42	*t* = 7.4, ***p*** **<** **0.001**, *r* = 0.62
Experiential avoidance	*t* = −4.4, ***p*** **<** **0.001**, *r* = 0.43	*t* = 6.8, ***p*** **<** **0.001**, *r* = 0.59
Expressive suppression	*t* = −5.0, ***p*** **<** **0.001**, *r* = 0.47	*t* = 7.4, ***p*** **=** **0.008**, *r* = 0.62
Worry	*t* = −10.1, ***p*** **<** **0.001**, *r* = 0.74	*t* = 11.5, ***p*** **<** **0.001**, *r* = 0.78

*Significant results are highlighted in bold text*.

### Person-Level ED and Within-Person Fluctuations

#### Differentiation Indicators

Negative ED was indexed by the ICC, which is a measure of the average consistency between the negative emotions. The ICC for negative emotions was obtained for each person (Demiralp et al., 2012; Erbas et al., 2018; Thompson et al., 2021). We excluded negative ICCs because these values are considered unreliable (Erbas et al., 2018). We transformed the remaining ICCs using a Fisher's Z transformation because ICCs are not normally distributed (cf. Barrett et al., 2001). To ease the interpretation of the indicators, we then reversed the Z-transformed ICC's, such that higher values indicate better differentiation. We calculated ED both at the day level (i.e., across measurement occasions within a specific day) and at the person level (i.e., across all measurement occasions (Erbas et al., 2018). For the day-level ED, we used person mean-centered variables, that is, daily within-individual fluctuations.

### Statistical Analysis

The first aim was addressed with correlation analyses between person-level ED and baseline ER strategies, exploring how person-level negative ED was associated with habitual use of individual ER strategies. MLMs were conducted to evaluate aims 2 and 3, where the repeated measurements (level 1) were nested within individuals (level 2). Specifically, either person-level ED (aim 2) or person mean-centered day-level ED (aim 3) served as the independent variable with employment of each of the daily ER strategies being the dependent variable in separate models. MLMs included a random intercept, and the repeated measure (i.e., the different observation occasions) was modeled with an “Autoregressive 1” covariance type. For MLMs with time-varying predictors (i.e., daily ED or ER), a random slope was specified for these. Following recent findings and recommendations, all analyses exploring ED–ER associations were run with and without negative emotions as a covariate, thereby exploring the extent to which ED is uniquely associated with ER, that is, above and beyond the level of negative emotions (see Dejonckheere et al., 2019; Kalokerinos et al., 2019). When exploring ED across the study period (between-person), the covariate included was the average level of negative emotions across the study period, and when exploring daily ED, the covariate referred to daily levels of negative emotions. Effect sizes were calculated as correlation coefficients (*r*), using *t*-to-*r* transformations (Kashdan and Steger, 2006; O'Toole et al., 2014), and values of 0.1, 0.3, and 0.5 were taken to denote a small, medium, and large effect size, respectively (Cohen, 1988). All analyses were performed in IBM SPSS Statistics Version 27.

## Results^2^

### Participants

One participant dropped out, resulting in 90 participants in total completing the survey. In sample 1, 88.2% of the participants were women, and the mean age was 23.7 (1.7) year. In Sample 2, 74.4% of participants were women, and the mean age was 25.4 (6.02) In total, 82.2 % were women, and the mean age was 24.4, ranging from 20 to 57. There was no statistical significant difference between the two samples regarding age (*p* = 0.106), education (*p* = 0.324), or gender (*p* = 0.088). Out of the total possible observations for each person, 22.1% responses were missing on average. Baseline means for emotional outcomes and ER strategies can be found in Table 4. Independent samples *t*-tests were used to compare scores between the two samples. Only one significant difference was detected. Sample 1 scored higher on rumination than Sample 2 [mean difference = 4.4, *t*_(86)_ = 2.1, *p* = 0.043]. No significant difference was detected for person-level negative ED, *t*_(86)_ = 0.3, *p* = 0.754. These results were taken as sufficient grounds for combining the two samples. Concerning missing ED data, it was not possible to calculate person-level ED for two participants (2%), and 248 daily records were missing out of 900 possible daily records. Of the remaining 652 records, 124 ICCs (19%) were deleted due to negative values.

**Table 4:** Participant descriptive statistics (*N* = 90).

	**Sample 1**	**Sample 2**	**Total**
	***M* (*SD*)**	***M* (*SD*)**	***M* (*SD*)**
**Emotions**			
Positive emotions	24.3 (5.2)	25.9 (4.0)	25.0 (4.8)
Negative emotions	13.9 (4.2)	13.3 (4.9)	13.6 (4.5)
**Putatively Adaptive Emotion Regulation**			
RRQ reflection	44.6 (10.0)	47.1 (8.3)	45.7 (9.3)
EQ decentering	37.8 (6.9)	39.2 (7.9)	38.4 (7.3)
FFMQ non-reactivity	21.3 (5.1)	22.0 (5.5)	21.60 (5.3)
ERQ reappraisal	29.5 (4.4)	30.5 (6.5)	29.9 (5.4)
**Putatively Maladaptive Emotion Regulation**			
ERQ expressive suppression	11.4 (5.1)	11.22 (5.1)	11.29 (5.1)
AAQ experiential avoidance	26.24 (5.9)	24.4 (6.1)	25.4 (6.0)
RRQ rumination	40.9 (9.7)	36.5 (10.6)	39.0 (10.2)
PSWQ worry	48.3 (11.7)	48.0 (9.6)	48.2 (10.8)

*Means (M) and Standard Deviations (SD) from baseline measures. Scores reflect mean total scores. AAQ, Acceptance and Action Questionnaire (Bond and Bunce, 2003); EQ, Experiences Questionnaire (Fresco et al., 2007); ERQ, Emotion Regulation Questionnaire (Gross and John, 2003), FFMQ, Five Facet Mindfulness Questionnaire (Baer et al., 2008); PSWQ, Penn State Worry Questionnaire (Meyer et al., 1990); RRQ, Rethinking Rumination Questionnaire (Nolen-Hoeksema et al., 2008)*.

### Associations Between Person-Level Negative ED and Habitual Use of ER Strategies

Turning to the first aim of investigating how person-level negative ED was associated with habitual use of individual ER strategies, correlation coefficients can be found in Table 2. Person-level negative ED showed positive and statistically significant correlations with habitual use of distancing and non-reactivity of a small to medium magnitude, and negative and statistically significant correlations with habitual use of rumination and worry, also of a small to medium magnitude. However, none of the ED–ER associations remained statistically significant after controlling for baseline negative emotions.

### Associations Between Person-Level and Person Mean-Centered Daily Negative ED and Daily Use of ER Strategies

For the second and third aim of exploring how person-level and person mean-centered daily negative ED were associated with daily use of individual ER strategies, mean total scores for daily measures can be found in Table 5, and results can be found in Table 6. For daily putatively adaptive ER strategies, person-level ED was positively associated with all ER strategies, but none of the associations were statistically significant when controlling for mean levels of negative emotions across the study period. For person mean-centered daily negative ED, the association with daily distancing was of a small to medium magnitude and remained borderline statistically significant after controlling for daily level of negative emotions.

**Table 5:** Mean total scores for daily emotions and emotion regulation strategies.

	***M* (*SD*)**
**Emotions**	
Positive emotions	18.0 (3.7)
Negative emotions	10.9 (2.2)
**Putatively Adaptive Emotion Regulation**	
RRQ reflection	4.9 (2.2)
EQ decentering	6.9 (1.7)
FFMQ non-reactivity	6.4 (1.7)
ERQ reappraisal	5.1 (2.2)
**Putatively Maladaptive Emotion Regulation**	
ERQ expressive suppression	4.6 (2.2)
AAQ experiential avoidance	4.1 (1.8)
RRQ rumination	4.8 (2.3)
PSWQ worry	3.5 (1.9)

*Emotion regulation strategy values reflect mean daily total scores of the two items (i.e., possible range from 2 to 10). AAQ, Acceptance and Action Questionnaire (Bond and Bunce, 2003); EQ, Experiences Questionnaire (Fresco et al., 2007); ERQ, Emotion Regulation Questionnaire (Gross and John, 2003), FFMQ, Five Facet Mindfulness Questionnaire (Baer et al., 2008); PSWQ, Penn State Worry Questionnaire (Meyer et al., 1990); RRQ, Rethinking Rumination Questionnaire (Nolen-Hoeksema et al., 2008)*.

**Table 6:** Results from separate multilevel models evaluating the association between daily emotion regulation strategies as a continuous measure and person-level and person mean-centered daily negative emotion differentiation (ED) (without/with daily negative emotions as a covariate).

	**Reflection**	**Distancing**	**Non-reactivity**	**Reappraisal**	**Rumination**	**Experiential avoidance**	**Expressive suppression**	**Worry**
Person-level negative ED	*t* = 0.6/0.2 *p* = 0.550/0.816 *r* = 0.06/0.02	*t* = 2.8/0.2 *p* = 0.006/0.840 *r* = 0.29/0.02	*t* = 2.6/0.2 *p* = 0.012/0.807 *r* = 0.27/0.03	*t* = 0.1/−0.1 *p* = 0.957/0.953 *r* = 0.01/0.01	*t* = −2.9/−0.4 *p* = 0.004/0.712 *r* = −0.30/−0.04	*t* = −2.4/−0.4 *p* = 0.019/0.655 *r* = 0.25/0.05	*t* = −1.1/0.4 *p* = 0.273/0.671 *r* = −0.12/0.04	*t* = −3.7/−1.3 *p* < 0.001/0.195 *r* = −0.36/−0.14
Person mean-centered daily negative ED	*t* = −1.2/−0.2 *p* = 0.235/0.852 *r* = −0.13/−0.02	*t* = 3.9/1.7 *p* = < 0.001/0.089 *r* = 0.39/0.18	*t* = 2.9/0.8 *p* = 0.005/0.410 *r* = 0.30/0.09	*t* = −0.9/0.4 *p* = 0.363/0.710 *r* = 0.10/−0.04	*t* = −3.0/−0.4 *p* = 0.004/0.792 *r* = −0.31/−0.03	*t* = −2.2/−0.1 *p* = 0.028/0.942 *r* = −0.23/−0.01	*t* = −2.5/−1.9 *p* = 0.014/0.061 *r* = −0.26/−0.20	*t* = −4.4/−1.1 *p* < 0.001/0.282 *r* = −0.43/−0.12

With regard to daily putatively maladaptive ER strategies, person-level ED was negatively associated with all strategies, but none of the associations were statistically significant when controlling for mean levels of negative emotions across the study period. For person mean-centered daily negative ED, the association with expressive suppression was of a small to medium magnitude and remained borderline statistically significant after controlling for daily level of negative emotions.

A set of *post-hoc* analyses was conducted, exploring the associations between ED and daily ER but categorizing ER categorically^3^. These analyses were conducted with the aim of approaching the research question in a similar manner but eliminating the inherent issue of including “1 s” in the average ER scores since those values represent the lack of use and not the amount of use. ER was rated as absent for scores assuming values of 1 and 2 and present for scores assuming values of 3 through 5 (i.e., mean total scores ≥6). Multilevel level logistic regression were run in Stata using the *melogit* command. The dependent variable was the categorical measure of ER use, with the independent variable being negative ED. A random intercept was specified in all models, and a random slope was specified for the time-varying predictor (i.e., within-person fluctuation in ED). Analyses were run with and without negative emotions. Results can be found in Table 7. None of the ED–ER associations were significant in models including negative emotion as a covariate. For both between-person negative ED (*r* range from <0.01 to 0.17) and within-person fluctuations in negative ED (*r* range from 0.1 to 0.15), all effect sizes were small.

**Table 7:** Results from separate multilevel models evaluating the association between daily emotion regulation strategies as a categorical measure (present or not present) and person-level and person mean-centered daily negative emotion differentiation (ED) (without/with daily negative emotions as a covariate).

	**Reflection**	**Distancing**	**Non-reactivity**	**Reappraisal**	**Rumination**	**Experiential avoidance**	**Expressive suppression**	**Worry**
Person-level negative ED	*z* = 0.31/0.37 *p* = 0.760/0.708 *r* = 0.03/0.04	*z* = 2.69/0.12 *p* = 0.003/0.901 *r* = 0.32/0.01	*z* = 2.43/0.29 *p* = 0.015/0.769 *r* = 0.26/0.03	*z* = 0.19/−0.04 *p* = 0.845/0.965 *r* = 0.02/ < −0.01	*z* = −2.67/0.16 *p* = 0.008/0.872 *r* = −0.28/0.02	*z* = −2.34/−0.38 *p* = 0.019/0.701 *r* = −0.25/−0.04	*z* = −1.32/0.80 *p* = 0.186/0.424 *r* = −0.14/0.09	*z* = −3.42/−1.62 *p* = 0.001/0.105 *r* = −0.35/−0.17
Person mean-centered daily negative ED	*z* = 1.06/1.42 *p* = 0.290/0.155 *r* = 0.11/0.15	*z* = 1.76/−0.36 *p* = 0.079/0.717 *r* = 0.19/−0.04	*z* = 2.46/0.60 *p* = 0.014/0.549 *r* = 0.26/0.06	*z* = −0.83/0.10 *p* = 0.408/0.923 *r* = −0.09/0.01	*z* = −2.09/0.79 *p* = 0.036/0.428 *r* = 0.22/0.08	*z* = −0.95/0.98 *p* = 0.343/0.326 *r* = −0.10/0.10	*z* = −1.09/−0.13 *p* = 0.276/0.897 *r* = −0.12/−0.01	*z* = −2.11/0.43 *p* = 0.035/0.668 *r* = −0.22/0.05

## Discussion

Negative ED has been suggested to be important for adaptive ER. However, knowledge concerning the association between ED and ER strategy choice is lacking. We wanted to add to this rather sparse literature on the ED–ER association, investigating how both person-level negative ED and within-person daily deviations from this person-level were associated with daily ER strategy endorsement. Consequently, we explored (1) how person-level negative ED was associated with habitual use of individual ER strategies, (2) how person-level negative ED was associated with daily use of individual ER strategies, and (3) how daily within-person fluctuations in negative ED were associated with daily use of individual ER strategies.

Concerning the association between person-level negative ED and habitual use of individual ER strategies, results were in the expected direction, showing that a higher score for negative ED (i.e., better differentiation abilities) was positively associated with greater use of the putatively adaptive strategies, and with less use of the putatively maladaptive strategies of rumination and worry. However, none of these associations, were statistically significant when controlling for mean levels of negative emotions. Regarding the associations between person-level negative ED and daily endorsement of ER strategies, a similar pattern was found with no statistically significant associations after controlling for mean level of negative emotions. Thus, true for both habitual and daily use of ER strategies, person-level negative ED did not show unique explanatory power above and beyond the mean level of negative emotions with all effect sizes being of negligible to small magnitudes (*r*s <0.15). These findings replicate the main conclusion reached by Kalokerinos et al. (2019), namely that person-level negative ED and ER strategy selection are only weakly associated.

Turning to the association between within-individual fluctuations in daily negative ED and daily ER strategies, associations also decreased in effect size and became non-significant when controlling for daily negative emotions. Thus, these results also largely confirms those obtained by Kalokerinos et al. (2019). Given a number of differences between studies, this may speak to the robustness of the overall finding of no or weak associations between ED and ER strategy endorsement. Study differences include that ER was operationalized at the momentary level in the study by Kalokerinos et al. (2019) and at the daily level in the present study. Moreover, ER efforts in the study by Kalokerinos et al. (2019) were evaluated “since the last beep,” where the present study inquired about ER efforts “in this current moment.” We chose the latter approach in an effort to obtain a measure directly tied to the moment in which the emotions occur. A time frame “since the last beep” may risk losing the specifics of the situations as multiple instances of ER could have happened since the last beep.

Concerning specifics of the situation, there appears to be strong consensus concerning the episodic nature of emotions, lasting seconds to minutes (e.g., Frijda, 1986; Gross, 2014). Indeed, theories and derived hypotheses surrounding ED often posit that the very reason that ED is adaptive is because the experienced emotions point to potential actions to be taken in a *particular situation* (e.g., Demiralp et al., 2012; Grühn et al., 2013; Kashdan et al., 2015). When calculating ED *across* assessment points (across the whole study period or across 1 day), one is left with an index that carries little information about the situational specifics. This could be argued to be appropriate when investigating overall associations between ED and general well-being or mental health (e.g., associations between ED and psychopathology; Smidt and Suvak, 2015) or changes in general ED skills over time (e.g., as ED may improve with psychotherapy; Van Der Gucht et al., 2019; Mikkelsen et al., 2021). However, it may not be considered appropriate when it comes to evaluating the potential effect of ED on choice of particular ER strategies calibrated to the particular situation. This has led some researchers to distinguish between trait and state ED (Tomko et al., 2015; O'Toole et al., 2020; Thompson et al., 2021), where “trait” refers to ED at the person level and calculated across a number of assessment points, and “state” refers to a single, momentary rating in a particular situation. Indeed, Tomko et al. (2015) argue that ED should be evaluated both at the trait and state level, claiming that disaggregating trait-level indicators into their state occurrences is a key step in understanding how ED influences behavior. Hence, the lack of significant associations between negative ED and ER choice identified in the present study may be a result of assessing ED across multiple measurement occasions as this produces an index that does not account for the calibration of ER to the specific situational contexts.

Concerning the empirical investigation of ED at the state level, research is still in its infancy. In a systematic review and meta-analysis, O'Toole et al. (2020) identified only two studies that had investigated the association between state ED on the one side and specific situational behaviors on the other (wise reasoning; Grossmann et al., 2016a; impulsivity; Tomko et al., 2015), both finding associations in the expected direction. More research of this type is needed to evaluate the effect of ED on ER at the situational level where emotions unfold and are believed to exert their influence. Furthermore, it would be important to empirically establish the causal relationship between ED and ER strategy choice, for which the theoretical assumption in the literature primarily points to a causal link *from* ED *to* ER (Kashdan et al., 2015; Thompson et al., 2021). Here, ED is believed to facilitate access to the information that emotions carry, making them less overwhelming and easier to regulate in a context-appropriate way (Barrett et al., 2001, Kashdan et al., 2010). However, an alternative possibility is that certain ER strategies exert an influence on ED, potentially facilitating better ED. Sometimes ER efforts may be employed specifically with the purpose of gaining a nuanced awareness of current emotions. Psychotherapies such as emotion regulation therapy (ERT; Mennin and Fresco, 2015) and acceptance-based behavior therapies (e.g., Roemer and Orsillo, 2009) teach clients a range of mindfulness-based ER strategies with which to gain such awareness. In these instances, improved ER skills (e.g., improved ability to use healthy strategies such as distancing over unhealthy strategies such as worry; Mennin and Fresco, 2015) may facilitate better emotion processing (Borkovec et al., 2004; Newman and Llera, 2011), which may in turn lead to improved ED (Mikkelsen et al., 2021). For the empirical investigation of ED at the state level, different analytic approaches have been developed. Derived from the person-level ED ICC, Erbas et al. (2021) introduce a momentary ED index, concerning fluctuations in ED relative to the person's overall level of ED. In addition, Grossmann et al. (2016a,b) have quantified state ED as a combined index of the number of experienced emotions (richness) and the relative intensity of each of the emotions (evenness). Finally, Tomko et al. (2015) have proposed yet another approach. They have estimated an individual's momentary ED by using variance decomposition analyses, in which negative affect subscale (e.g., for fear, hostility, sadness) and items per subscale (e.g., 5 items per subscale) are entered as sources of variability (i.e., factors) in an ANOVA model. From this model, researchers can derive an indicator of ED capturing the consistency of ratings across negative affect subscales in a given moment.

As for the conceptualization of ER strategies as putatively adaptive or maladaptive, the detected associations between person-level ER strategies and emotions were largely as expected. The same was true when investigating person mean-centered ER with the exception of reappraisal, which was positively associated with negative emotions. Such associations could point to the emotional effect of ER strategies, however, they could also reflect that individuals are more prone to use certain strategies over others in response to either positive or negative emotions.

The present results should be viewed in light of important limitations. First, the number of daily prompts was relatively low (i.e., 3 and 4 prompts) and past studies have suggested to calculate daily ICCs only for individuals with a higher number of completed prompts per day (e.g., 6 ESM prompts per day, Erbas et al., 2018). This small number of prompts may pose a threat to the reliability of the daily ICCs. Further in regard to the ICC, we opted to delete negative ICCs. Other approaches have sometimes been used in the literature such as retaining negative ICCs by forcing them to assume a value of 0 (Thompson et al., 2021). Second, the sample size was relatively small (*n* = 90) and this number was not determined *a priori* with the present analyses in mind. In terms of statistical power, we were not able to detect effect sizes of a small to medium magnitude (*r* = 0.20) as statistically significant in the full MLM controlling for negative emotions. Future studies could look to this effect size for the determination of their sample size. Third, two samples were combined in order to increase sample size and thereby statistical power. Although they were largely similar concerning baseline characteristics, it cannot be ruled out that the slight difference in procedures may have affected the investigated ED–ER association. In addition, while the validity (i.e., association between the full ER scale at baseline and the daily scores) and reliability (i.e., within-person correlation between the two daily items across the study period) were generally acceptable, two exceptions should be noted. Validity was low for the reappraisal items, although coming close to the pre-determined criterion (0.27 vs. 0.30). In this regard, it should be mentioned that mixed results have previously been found concerning the strength of the association between trait and daily measures of ER (McMahon and Naragon-Gainey, 2020). This could both point to habitual or trait ER being differentially associated with daily ER depending on the strategy, or to measurement error in the daily measures with only few items (i.e., often only one item and in the present study two). Furthermore, reliability was low for the items pertaining to distancing and experiential avoidance. Such finding may reflect different facets of the ER strategies being measured or even different ER strategies altogether. However, although not meeting the *r* ≥ 0.5 criterion, the items were moderately correlated (*r*s ≥ 0.3). Finally, positive ED and other measures of emotion experience complexity (e.g., emotion covariation, emotion variability; Grühn et al., 2013) were not evaluated, leaving it unclear how the findings extend to such.

In conclusion, the results of the present study add to the sparse literature concerning the link between ED and ER. The present findings indicate weak or no associations between ED and ER strategy endorsement when controlling for negative emotions. Experimental research addressing ED at the momentary level and teasing out the causal relationship between ED and choice of ER strategies is needed to gain further insight into this matter. Such insight may represent an important step toward refining psychotherapeutic interventions aimed at improving emotion problems.

## Data Availability Statement

The datasets presented in this study can be found in online repositories. The names of the repository/repositories and accession number(s) can be found below: doi: 10.17632/bnxjdwhzh6.1.

## Ethics Statement

Ethical review and approval was not required for the study on human participants in accordance with the local legislation and institutional requirements. The patients/participants provided their written informed consent to participate in this study.

## Author Contributions

EE was responsible for preparing and conducting the experiments, MO'T and MM for analyzing the resulting data, and MO'T for writing the initial draft of this paper. All authors developed and formulated the overarching research goals, contributed to the article, and approved the submitted version. The authors state that they have reported all measures, conditions, data exclusions, and reflections on sample sizes.

## Conflict of Interest

The authors declare that the research was conducted in the absence of any commercial or financial relationships that could be construed as a potential conflict of interest.

## Footnotes

^1^Additional measures that were included in Study 1 at baseline were the Positive and Negative Affect Schedule (Watson et al., 1988), the Hospital Anxiety and Depression Scale (Bjelland et al., 2002), and the Satisfaction With Life Scale (Diener et al., 1985). At the daily level, additional measures included the Depicted Action Tendencies (O'Toole and Mikkelsen, 2021), single item rating of well-being, and two items concerning current activities. In Study 2, Toronto Alexithymia Scale (TAS-20; Bagby et al., 1994) was included at baseline. Data from these questionnaires were not analyzed for the present study.

^2^The data have been used in another study (Elkjær et al., 2021) where the main aim was to explore emotion regulation flexibility. There are no analytic overlaps between that and the present study.

^3^These analyses were suggested in the review process and were thus not determined *a priori*.
